# β-Catenin Knockdown Affects Mitochondrial Biogenesis and Lipid Metabolism in Breast Cancer Cells

**DOI:** 10.3389/fphys.2017.00544

**Published:** 2017-07-27

**Authors:** Daniele Vergara, Eleonora Stanca, Flora Guerra, Paola Priore, Antonio Gaballo, Julien Franck, Pasquale Simeone, Marco Trerotola, Stefania De Domenico, Isabelle Fournier, Cecilia Bucci, Michel Salzet, Anna M. Giudetti, Michele Maffia

**Affiliations:** ^1^Department of Biological and Environmental Sciences and Technologies, University of Salento Lecce, Italy; ^2^Laboratory of Clinical Proteomic, “Giovanni Paolo II” Hospital Lecce, Italy; ^3^CNR NANOTEC - Institute of Nanotechnology Lecce, Italy; ^4^University of Lille, Institut national de la santé et de la recherche médicale, U-1192 - Laboratoire Protéomique, Réponse Inflammatoire et Spectrométrie de Masse-PRISM Lille, France; ^5^Unit of Cytomorphology, CeSI-MeT and Department of Medicine and Aging Sciences, School of Medicine and Health Sciences, University “G. d'Annunzio” Chieti, Italy; ^6^Unit of Cancer Pathology, CeSI-MeT and Department of Medical, Oral and Biotechnological Sciences, University “G. d'Annunzio” Chieti, Italy; ^7^C.N.R. Unit of Lecce, Institute of Food Production Sciences Lecce, Italy

**Keywords:** β-catenin, proteomics, LC-MS/MS, mitochondria, lipid metabolism, MYC

## Abstract

β-catenin plays an important role as regulatory hub in several cellular processes including cell adhesion, metabolism, and epithelial mesenchymal transition. This is mainly achieved by its dual role as structural component of cadherin-based adherens junctions, and as a key nuclear effector of the Wnt pathway. For this dual role, different classes of proteins are differentially regulated via β-catenin dependent mechanisms. Here, we applied a liquid chromatography-mass spectrometry (LC-MS/MS) approach to identify proteins modulated after β-catenin knockdown in the breast cancer cell line MCF-7. We used a label free analysis to compare trypsin-digested proteins from CTR (shCTR) and β-catenin knockout cells (shβcat). This led to the identification of 98 differentially expressed proteins, 53 of them were up-regulated and 45 down-regulated. Loss of β-catenin induced morphological changes and a significant modulation of the expression levels of proteins associated with primary metabolic processes. In detail, proteins involved in carbohydrate metabolism and tricarboxylic acid cycle were found to be down-regulated, whereas proteins associated to lipid metabolism were found up-regulated in shβcat compared to shCTR. A loss of mitochondrial mass and membrane potential was also assessed by fluorescent probes in shβcat cells with respect to the controls. These data are consistent with the reduced expression of transcriptional factors regulating mitochondrial biogenesis detected in shβcat cells. β-catenin driven metabolic reprogramming resulted also in a significant modulation of lipogenic enzyme expression and activity. Compared to controls, β-catenin knockout cells showed increased incorporation of [1-^14^C]acetate and decreased utilization of [U-^14^C]glucose for fatty acid synthesis. Our data highlight a role of β-catenin in the regulation of metabolism and energy homeostasis in breast cancer cells.

## Introduction

β-catenin is a multifunctional protein localized at multiple subcellular regions, including adherents junctions, cytoplasm and/or nucleus. β-catenin plays an essential role in the maintenance of adult tissue homeostasis. In normal epithelial cells, β-catenin is mainly localized at the plasma membrane, where it interacts with the cytoplasmic tail of E-cadherin to form a cell adhesion complex at the intercellular junctions (Huber and Weis, 2001; Tian et al., 2011). In normal breast tissues, β-catenin is highly expressed at the basolateral surface of the luminal epithelium, as compared to the lateral surface of the myoepithelial cells, where it is expressed at much lower levels (Hashizume et al., 1996).

Dysregulation of the β-catenin/E-cadherin complex can induce profound defects in the organization of epithelial layers leading to development of mammary tumors, among them adenocarcinoma and squamous metaplasia (Teulière et al., 2005). Disruption of cadherin-mediated cell adhesion has been proposed to drive the epithelial-mesenchymal transition (EMT) program, a process occurring in development and cancer, whereby epithelial cells lose cell-cell contacts and apical-basal polarity, and acquire a mesenchymal-like morphology (Thiery and Sleeman, 2006; Lamouille et al., 2014).

The Wnt pathway regulates β-catenin signaling, and nuclear localization of β-catenin is a hallmark of the activation of Wnt pathway. In the absence of Wnt ligands, the cytoplasmic pool of β-catenin protein undergoes proteasomal degradation (MacDonald et al., 2009), thus preventing β-catenin localization into the nucleus. In this way, Wnt/β-catenin target genes are repressed by the DNA-bound T cell factor/lymphoid enhancer factor (TCF/LEF) and transducing-like enhancer (TLE/Groucho) protein complex (MacDonald et al., 2009). In the canonical Wnt pathway, binding of secreted Wnt ligands to Frizzled receptors and lipoprotein receptor-related proteins (LRP) co-receptors stimulates the stabilization of the cytosolic β-catenin pool by phosphorylation, leading to β-catenin translocation and transcriptional regulation of target genes (Tetsu and McCormick, 1999; Cong et al., 2004). Aberrant activation of the canonical Wnt/β-catenin pathway is frequently observed in several human cancers. Wnt pathway components, including Axin, β-catenin and Adenomatous polyposis coli (APC), are frequently mutated in colorectal adenocarcinoma but this is uncommon in breast cancer samples (Zhan et al., 2017), where β-catenin activation is not driven by β-catenin gene (*CTNNB1*) mutations (Geyer et al., 2011). β-catenin aberrant nuclear signaling was observed in Her2 and triple-negative/basal-like breast carcinomas and associated with cancer progression and poor clinical outcome (Geyer et al., 2011; Schade et al., 2013). In ErbB2-positive breast cancer, reduced β-catenin levels lead to the activation of an EMT program characterized by down-regulation of adherens junctions and sustained nuclear localization of β-catenin (Tung et al., 2017). Suppression of Wnt/β-catenin signaling results in reduced migration *in vitro* and *in vivo*, reduced tumor growth, and reduced expression of stem cell markers (Jang et al., 2015).

Overall, these findings depict a key role of β-catenin in the regulation of tumor initiation and progression, stemness, proliferation and invasion. However, the substantial concept of β-catenin as regulator of development and stemness has now expanded, and β-catenin is found to be involved in a large variety of cellular processes. Analysis of KEGG pathways/functions revealed that up- or down-regulations of β-catenin lead to altered regulation of actin cytoskeleton, insulin signaling and metabolism (Herbst et al., 2014), though the molecular mechanisms underlying these associations appear still unclear. In this scenario, the complexity of the β-catenin network can be fully addressed by experimental approaches that allow managing thousands of molecules simultaneously.

Here, we performed a liquid chromatography-mass spectrometry (LC-MS/MS) analysis to characterize the proteomic modifications associated with the stable knockdown of β-catenin in the breast cancer cell line MCF-7. By integrating label-free MS, bioinformatics pathway analysis, and cell-based experimental approaches we identified 98 significantly modulated proteins associated with distinctive molecular functions and cellular processes. Specifically, we gained insight into the mechanisms that link β-catenin to modifications of metabolic proteins, and we described in our model a metabolic reprogramming accompanied by alterations in mitochondrial function and lipid metabolism.

## Materials and methods

### Cell culture and reagents

Human tumor cells were purchased from Banca Biologica and Cell Factory (IRCCS Azienda Ospedaliera Universitaria San Martino-IST Istituto Nazionale per la ricerca sul cancro, Genova, Italy). The human cancer cell line MCF-7 was cultured in high glucose DMEM (Euroclone) supplemented with 10% FBS (Euroclone), 100 U/ml penicillin and 100 μg/ml streptomycin at 37°C in an atmosphere of 5% CO_2_. The cells were maintained in an exponential growth phase during experiments.

Epidermal growth factor (EGF) was from Prospec. Primary antibodies were from Santa Cruz: β-Catenin (sc-1496), α-Tubulin (sc-23948), Cofilin (sc-33779), phospho-Cofilin (sc-21867-R); or Cell Signaling: β-Actin (#8457), c-Myc (#13987), fatty acid synthase (FASN, #3180), and Acetyl CoA Carboxylase (ACC, #3676). Secondary antibodies (HRP-conjugated) were from Santa Cruz Biotechnology (goat anti-mouse IgG-HRP, sc-2005; goat anti-rabbit IgG-HRP, sc-2004) or Cell Signaling (anti-rabbit IgG-HRP, #7074). Phalloidin-tetramethylrhodamine B isothiocyanate (TRITC) and Fluoroshield were from Sigma. All other reagents were from standard commercial sources and were of the highest grade available.

### β-catenin silencing

To obtain MCF7 cells with stable knockdown of endogenous β-catenin, lentiviral particles were generated using HEK-293T cell line and the pLKO.1 puro shRNA β-catenin vector (shβcat) (a gift from R. A. Weinberg, Addgene plasmid #18803) (Onder et al., 2008) as previously described (Trerotola et al., 2012). Non-silencing shRNA in pLKO.1 puro vector was a gift from D. Gabellini, and was used as negative control (shCTR). Lentiviruses were used to infect MCF7 cells. Stably silenced cells were obtained by selection with puromycin at 10 μg/ml for 2 weeks. Western Blotting and Real time PCR were carried out to assess down-regulation of β-catenin.

### Cell proliferation assay

Cells were plated at 1 × 10^4^/well in a 96-well plate and allowed to adhere to the plate overnight. Cell proliferation was then determined using the 3-(4, 5-dimethylthiazolyl-2)-2,5-diphenyltetrazolium bromide (MTT) assay at different time points. Briefly, the culture medium was removed and 100 μl of RPMI-phenol free medium containing 10 μl of MTT stock solution, 5 mg/ml in phosphate-buffered saline (PBS) solution, were then added to each well. After 1 h incubation, the MTT solution was removed and 100 μl of DMSO were added to solubilize MTT-formazan crystals. Absorbance of the converted dye was measured at 570 nm using an iMark microplate reader (Biorad).

Cells were cultured in 24-well plate until confluence and then wounded using a 200 μl pipette tip in the middle of well. Three wounds were made for each sample, and migration distance was measured at time zero and after 24 and 48 h of stimulation with EGF (medium was replaced every day), under an Olympus IX-51 microscope. The percentage (%) of open wound area was determined and the change in open wound area (%) at 24 and 48 h against zero time was calculated using the GraphPad PRISM software version 4.0.

### Sample preparation and mass spectrometry analysis

Whole protein extraction was carried out using the Illustra TriplePrep kit (GE Healthcare) and samples were then processed according to the filter-aided sample preparation (FASP II) protocol (Wiśniewski et al., 2009). Briefly, approximately 20 μg of protein extract were diluted tenfold in 8 M urea in 0.1 M Tris/HCl pH 8.5, loaded into the Microcon Ultracel YM-30 filtration devices (Millipore), and centrifuged at 14,000 × g for 15 min. The concentrates were then diluted in 8 M urea and centrifuged again. After centrifugation, proteins were reduced in 10 mM dithiotreitol (DTT) for 30 min, and then alkylated in 50 mM iodoacetamide (IAM) for 20 min. After 4 washes (2 in 8 M urea and 2 in 50 mM NH_4_HCO_3_), trypsin solution was added to the filter (enzyme-to-protein ratio 1:100 w/w), and samples were incubated at 37°C overnight. Peptides were collected by centrifugation followed by an additional wash with 50 mM NaCl. Finally, the peptide mixture was acidified by trifluoroacetic acid, desalted-concentrated on C-18 ZipTip (Millipore), dried under vacuum and then resuspended in 20 μL of ACN/H2O (FA 0.1%) (2:98, v/v).

Separation was performed using an EASY-nLC 1000 UPLC (Thermo Scientific) equipped with a 75 μm × 2 cm pre-column with nanoViper fittings (Acclaim pepMap 100, C18, 2 μm, Thermo Scientific) and a 50 μm ID × 150 mm analytical column with nanoViper fittings (Acclaim PepMap RSLC, C18, 2 μm, Thermo Scientific). Elution was carried out using a 2-h gradient of ACN starting from 5 to 30% over 120 min at a flow rate of 300 nl/min. The Q-Exactive instrument (Thermo Scientific) was set to acquire top 10 MS2 with a spray voltage of 1.6 kV. The survey scans were taken at 70,000 FWHM (at m/z 400) resolving power in positive ion mode and using a target of 3 E6 and default charge state of +2. Unassigned and +1 charge states were rejected, and dynamic exclusion was enabled for 20 s. The scan range was set to 300–1,600 m/z. For the MS2, 1 microscan was obtained at 17,500 FWHM and isolation window of 4.0 m/z, using a first mass at m/z 140.

### Database searching and bioinformatics analysis

Raw files obtained from nanoLC-MS were processed using the MaxQuant proteomics software (version 1.5.3.8) (Cox and Mann, 2008) as described (Duhamel et al., 2015). Q-Exactive spectra were matched to peptide sequences in the human UniProt protein database (release November 2014, 88,876 entries) using the Andromeda algorithm (Cox et al., 2011). Trypsin was used as enzyme and two missed cleavages were allowed. N-terminal acetylation and methionine oxidation were selected as variable modifications, and carbamidomethylation of cysteines was set as a fixed modification. For the MS spectra, an initial mass accuracy of 6 ppm was selected, and the MS/MS tolerance was set to 20 ppm for the HCD data. False discovery rate was set to 1% for peptides and proteins identification. Relative, label-free quantification of the proteins was conducted using the MaxLFQ algorithm (Cox et al., 2014).

Statistical analysis was performed with the Perseus software (version 1.5.2.4). Hierarchical clustering on Z-scored values was based on Euclidean distance and average linkage clustering. Differentially expressed proteins were analyzed according to their molecular function and biological process by Protein Analysis Through Evolutionary Relationships (PANTHER) software (version 11.0, http://www.pantherdb.org) (Mi et al., 2013). Gene ontology (GO) characterization was performed in PANTHER database using gene name list of differential expression protein.

### RNA extraction and real time PCR

Total RNA was extracted from cells grown in a T25 flask using the Illustra triplePrep kit (GE Healthcare). The reverse transcriptase reaction (20 μl) was carried out using 1 μg of total RNA, oligo(dT)18 and 200 units of SuperScriptTM III RNase H-Reverse transcriptase (Invitrogen) according to the manufacturer's protocol. Quantitative gene expression analysis was performed in a 7,300 Real time PCR System (Applied Biosystem) using iTaq Universal SYBR Green supermix (Biorad). Primers used in Real time PCR were reported in Table 1. The efficiency of each primer was tested running a standard curve in duplicate. The quantifications were performed using the ΔΔCT method and *Rplp0* gene was used as an internal control for normalization. Results are expressed as % of control. The specificity of PCR products was confirmed by melting curve analysis and agarose gel electrophoresis.

**Table 1:** Oligonucleotides used for Real time PCR analysis.

**Gene**	**Accession number**	**Primer sequence 5′-3′**	**bp**
*CTNNB1*	NM_001098209.1	βcat F: AACTTGCCACACGTGCAATC βcat R: GCGGTACAACGAGCTGTTTC	205
*CCND1*	NM_053056	CyclinD F: CGCTTCCTGTCGCTGGAGCC CyclinD R: CTTCTCGGCCGTCAGGGGGA	111
*MYC*	NM_002467.4	MYC F: CGACTCTGAGGAGGAACAAG MYC R: GTGCTGATGTGTGGAGACG	172
*SREBF1*	NM_001005291.2	SREBP F: AGCGTCTACCATAGCCCTG SREBP R: TGGCTCACCGTAGACAAAG	153
*FASN*	NM_004104	FASN F: CCTGCGTGGCCTTTGAAAT FASN R: CATGTCCGTGAACTGCTGC	204
*ACACA*	NM_198839	ACC F: GATCAAGGTCAGCTGGTCC ACC R: AACAAATCCTCTTGAGGCC	174
*ACLY*	NM_001303274.1	ACLY F: AGGGAGTGACCATCATCGGA ACLY R: GGTACCTGTCCCCACCAATG	231
*SLC25A1*	NM_001256534.1	CiC F: TGCTGCAGGAACGACCAGGA CiC R: CACGGTCTCCATGGGAATC	189
*CAV1*	NM_001753.4	CAV1 F: CGTAGACTCGGAGGGACATC CAV1 R: GTGTTTAGGGTCGCGGTTGA	156
*CD36*	NM_000072.3	CD36 F: ATGCAGCCTCATTTCCACC CD36 R: AGGCCTTGGATGGAAGAAC	150
*SLC2A1*	NM_006516.2	Glut1 F: GGCCATCTTTGGCTTTGTG Glut1 R: TAGGGACCACACAGTTGCTC	186
*T-FAM*	NM_001270782.1	TFAM F: CCGAGGTGGTTTTCATCTGT TFAM R. ACGCTGGGCAATTCTTCTAA	147
*NRF1*	NM_005011.3	NRF1 F: CCGTTGCCCAAGTGAATTAT NRF1 R: ACTGTAGCTCCCTGCTGCAT	181
*PPARGC1A*	NM_013261.3	Pgc1α F: GCTGACAGATGGAGACGTGA Pgc1α R: TGCATGGTTCTGGGTACTGA	178
*Rplp0*	NM_001697.2	36B4 F: TCGACAATGGCAGCATCTAC 36B4 R: ATCCGTCTCCACAGACAAGG	191
*Dloop*	AC_000022.2	Dloop F: GGTTCTTACTTCAGGGCCATC Dloop R: TGACCTTCATGCCTTGACGG	201
*Gapdh*	NG_007073.2	Gapdh F: ATGCCTTCTTGCCTCTTGTC Gapdh R: CATGGGTGGAATCATATTGG	245

### Western blot analysis

Whole proteins were extracted in RIPA buffer (Cell Signaling) and quantified by the Bradford protein assay (Biorad). Samples were separated by 12% SDS–PAGE and transferred to Hybond ECL nitrocellulose membranes (GE Healthcare). The membranes were blocked with Blotto A (Santa Cruz) at room temperature for 1 h, and incubated with the appropriate primary antibodies for 2 h at room temperature, as previously described (Vergara et al., 2016). After two washes with a solution of TBS containing 0.1% (v/v) tween 20 for 10 min, the membranes were incubated with secondary antibody HRP-conjugated for 2 h at room temperature (standard dilution 1:2,000). Blots were then developed using the Amersham ECL western blotting detection system (GE Healthcare). Densitometric quantitation of at least three independent replicates was done using ImageJ software.

### Lipogenic measurements

Lipogenic activity was determined by monitoring the incorporation of [1-^14^C]acetate (0.96 mCi/mmol) or [U-^14^C]glucose (0.2 mCi/ml) into fatty acids. Fresh medium was added together with labeled substrate 1 h before ending the incubations. At the end of the incubation time, the medium was removed and the adherent cells were washed three times with ice-cold 0.14 M KCl to remove the unreacted labeled substrate, and the reaction was stopped with 1.5 ml of 0.5 N NaOH. Cells were scraped off with a rubber policeman and transferred to a test tube. Fatty acids were extracted and counted for radioactivity as reported (Giudetti et al., 2013).

### Assay of acetyl-CoA carboxylase

The activity of acetyl-CoA carboxylase was determined as the incorporation of radiolabelled acetyl-CoA into fatty acids in a coupled assay with fatty acid synthase (FASN) reaction in digitonin-permeabilized cells as described previously (Priore et al., 2007). Reactions were carried out at 37°C for 8 min. After saponification for 30 min at 100°C and acidification with 7N HCl, labeled fatty acids were extracted with petroleum ether and, after evaporation of the ether phase, radioactivity was counted.

### Confocal microscopy analysis

For confocal microscopy analysis, MCF-7 and MCF-7/shβcat cells were grown onto glass coverslips at 4 × 10^6^ cells/ml in 6-well plates overnight. Seeded cells were then fixed for 10 min in 4% paraformaldehyde. For F-actin staining, fixed cells were washed twice with PBS and incubated with phalloidin-TRITC (P1951, Sigma) according to the manufacturer's protocol. α-Tubulin (tub) and β-catenin stainings were carried out following the manufacturer's protocol (Santa Cruz). Afterwards, samples were incubated with Alexa Fluor 488 (AF488)-conjugated secondary antibody (Cell Signaling). Slides were cover-slipped using a mounting medium containing 4′6-diamidino-2-phenylindole (DAPI) in order to counterstain nuclei (F6057, Sigma). The micrographs of fluorescently labeled proteins were acquired using a confocal laser scanning microscope (CLSM) (TCS SP5; Leica, Microsystem GmbH, Mannheim, Germany) equipped as in Vergara et al. (2015). DAPI fluorescent signal (in blue) was revealed with a 415–500 nm band pass filter, AF488-labeled secondary antibody (in green) was detected with a 495–519 nm band pass filter, and TRITC-phalloidin (in red) was evidenced with a 565–660 nm band pass filter. Images were taken with a HCX PL APO lambda blue 63.0 × 1.40 oil-immersion objective under sequential mode acquisition (scan mode: xyz; scan speed: 200 Hz). The pinhole aperture was set to 1 Airy.

### Live confocal fluorescence microscopy

Cells were seeded into sterile microscopy chambers (8 well μ-slide, IBIDI) at a density of 20,000 cells/well. After 24 h, for mitochondria staining, the cells were incubated with MitoTracker Red CMXRos (M7512, ThermoFisher Scientific; 1 mM) for 45 min at 37°C and with MitoTracker Green FM (M7514, ThermoFisher Scientific; 1 mM) and washed 3 times with PBS. After 3 washes in PBS, L-15 medium (Leibowitz medium without phenol red, Invitrogen) was added and the cells were imaged by confocal live microscopy. Emission intervals for individual dyes were: MitoTracker Red CMXRos: 560–615 nm (λ_ex_ = 555 nm) and MitoTracker Green FM: 510–550 nm (λ_ex_ = 488 nm). The images of fluorescently labeled proteins were captured using a confocal laser scanning microscope (CLSM) (Zeiss, LSM 700, Germany) equipped with a laser diode emitting at 405 nm, an argon-ion laser for excitation at 488 nm, and a helium-neon laser for excitation at 514 nm. Images were taken with a Plan - Apochromat 63.0 × 1.40 oil-immersion objective and the pinhole aperture was set to 1 Airy.

### Determination of mtDNA copy number

Total DNA from cells was obtained by phenol/chloroform extraction. Quantitative Real time PCR was performed to quantify mitochondrial DNA (mtDNA) content. The primers used for Dloop and Gapdh, mitochondrial and nuclear specific DNA sequences respectively, are reported in Table 1. mtDNA level was expressed as the ratio of mtDNA to nuclear DNA quantity (mtDNA/nDNA) (Serviddio et al., 2014).

### Statistical analysis

Data were expressed as mean ± SD. Statistical analyses was determined by paired Student's *t*-test. In all comparisons, *p* < 0.05 was considered as statistically significant.

## Results

### β-catenin knockdown induces morphological and functional changes in MCF-7 cells

**Figure 1:**
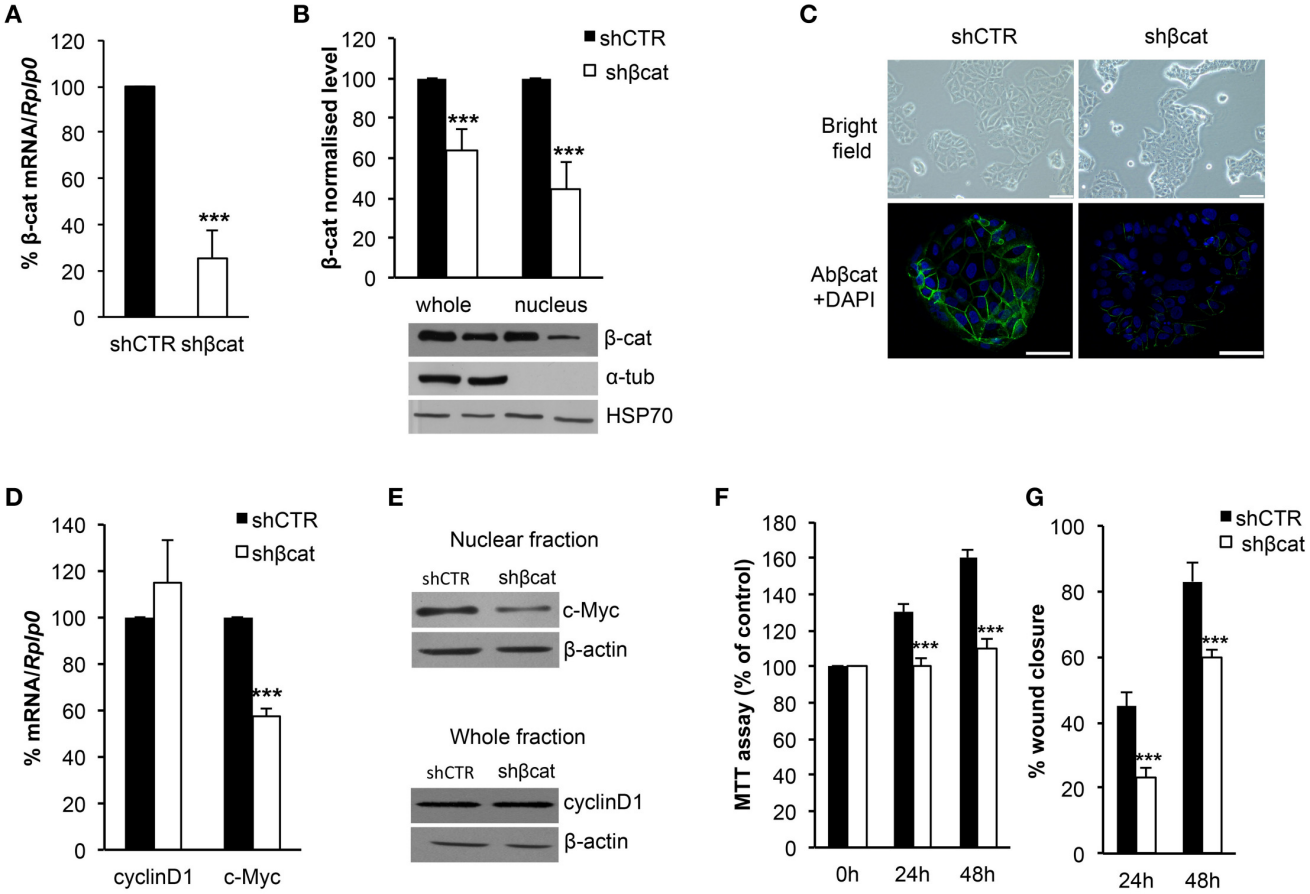
Evaluation of β-catenin silencing efficiency. β-catenin (β-cat) silencing efficiency was measured by **(A)** Real time PCR and **(B)** Western blotting. β-cat mRNA and protein levels were expressed as percentage of control. The protein content in the nuclear and cytoplasmic fraction was quantified by densitometric analysis of blots, α-Tub was used to exclude a contamination with cytoplasmatic proteins. HSP70 was used as a nuclear fraction loading control. Data represent the mean ± SD from 3 independent experiments, ***P < 0.001. **(C)** Upper panel: images taken with an Olympus IX51 inverted microscope (10× magnification) showing MCF-7 shCTR and MCF-7 shβcat cells (scale bars, 12.5 μm); lower panel: CLSM micrographs of β-cat (stained with β-cat, in green) in MCF-7 shCTR and MCF-7 shβcat cells (nuclei stained with DAPI, in blue; scale bars, 25 μm). **(D)** Real time PCR was used to determine mRNA expression levels of c-Myc and cyclinD1. *Rplp0* was used as housekeeping gene and the levels were expressed as percentage of control. Data represent the mean ± SD from 3 independent experiments, and ***P < 0.001. **(E)** Nuclear fraction protein was immunoblotting with c-Myc and whole fraction protein was immunoblotting with cyclinD1. β-actin was used for signal normalization. **(F)** MCF-7 shCTR and MCF-7 shβcat cells were seeded at 1 × 10^4^/well in a 96-well plate and incubated for 24 h and 48 h before estimating the cell proliferation rate by MTT test (***P < 0.001). **(G)** The percentage (%) of open wound area at 24 and 48 h against zero time was calculated using the GraphPad PRISM software version 4.0. Data represent the mean ± SD from 3 wounds for each sample, ***P < 0.001.

**Figure 2:**
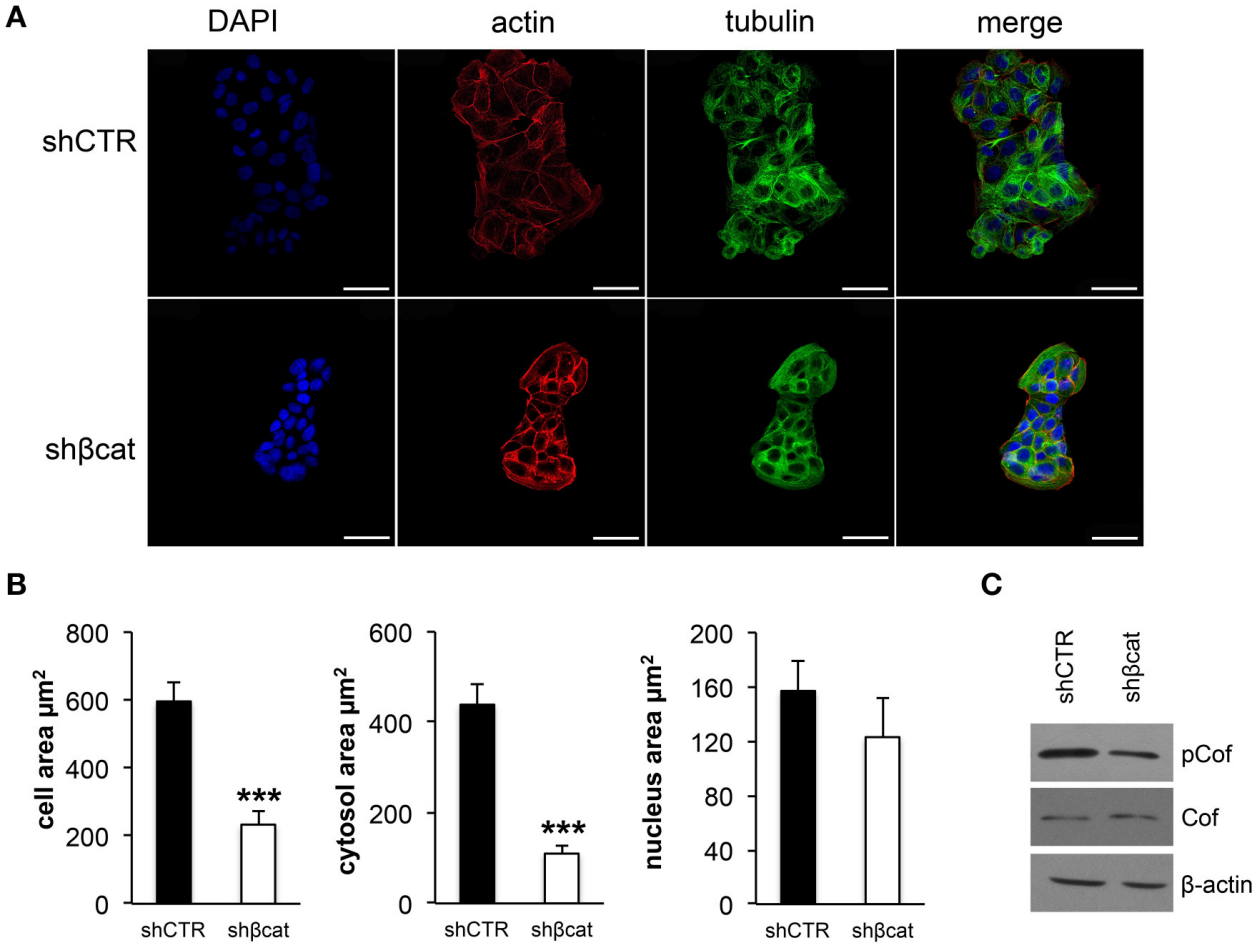
Effect of β-catenin gene silencing on morphology of MCF-7 cells. **(A)** CLSM micrographs of organization of F-actin (stained with phalloidin, in red) and microtubules (stained with α-tub, in green) filaments in MCF-7 shCTR and MCF-7 shβcat cells (nuclei stained with DAPI, in blue). The individual blue, red and green channels are shown followed by merged images (scale bars, 25 μm). The images are representative of three independent experiments. **(B)** Averages of cell, cytosol and nucleus areas of MCF-7 shCTR and s MCF-7 hβcat cells. Columns represent mean ± standard deviation (*n* cell analyzed = 50). ***P < 0.001. The outlines of DIC images of single cells and single nuclei were used for morphometric analysis. ImageJ64 was used for entering and storing the cell and nucleus outlines and for calculating the cellular and nuclear areas. **(C)** Western blot of phospho-Cofilin (pCof) and Cof protein levels. β-actin was used for signal normalization. The images are representative of three independent experiments.

**Figure 3:**
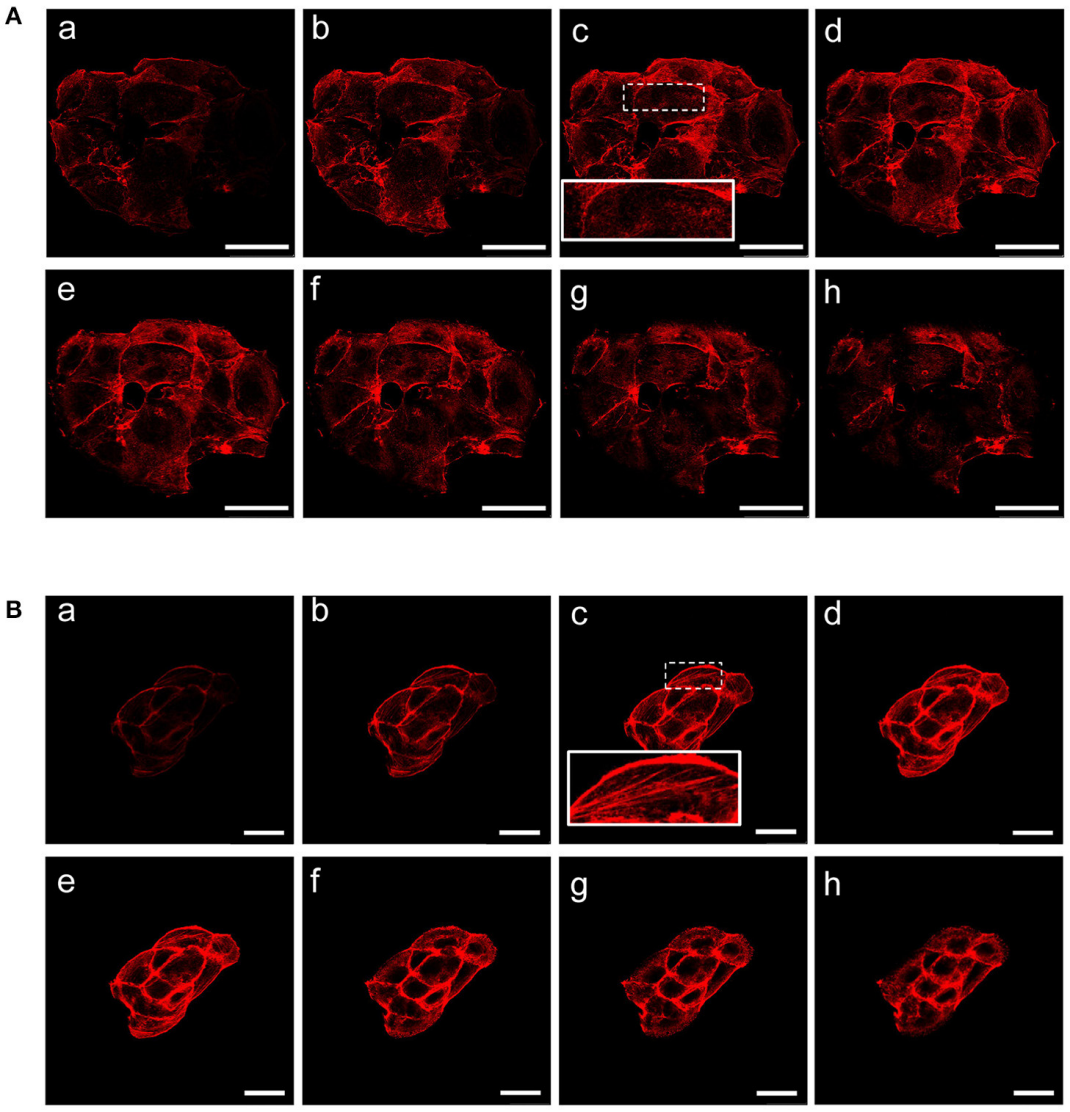
β-catenin gene silencing alters the organization of actin stress fibers. Z stacks of confocal images from the the basal to the apical sides **(a–h)** (depth interval = 0.21 μm) of MCF-7 shCTR **(A)** and MCF-7 shβcat cells **(B)** stained for F-actin. Scale bars, 50 μm **(A)** and 25 μm **(B)**. The images are representative of three independent experiments.

We sought to investigate the molecular and functional changes associated with β-catenin knockdown. Thus, we stably infected MCF-7 cells with lentiviruses for the expression of specific β-catenin shRNAs. Real time PCR, Western blot and fluorescence microscopy were used to validate efficient knockdown of β-catenin (Figures 1A–C). Overall, these experiments confirmed the reduced expression level of β-catenin at mRNA and protein level and validated our experimental model. In detail, β-catenin expression is reduced at membrane and nuclear level and became barely detected at cell-cell junctions. We characterized morphological changes by phase-contrast microscopy and confocal microscopy. Phenotypically, we noticed that shβcat cells show remarkable morphological changes as compared with shCTR cells. Inverted microscopic analysis revealed that shβcat cells lose polygonal-shape morphology with a consequent cell volume reduction (Figure 1C). To confirm functional effect on β-catenin associated transcriptional program, we analyzed the expression level of selected β-catenin target genes. As shown in Figures 1D–E, β-catenin down-regulation significantly reduced c-Myc expression, but did not result in a significant reduction of cyclin D1. As a functional consequence of all these alterations, shβcat cells displayed a reduced proliferation and wound closure after EGF stimulation compared to shCTR (Figures 1F,G). Cadherin/Catenin adhesion molecules function as a membrane-spanning macromolecular complex that interacts in a dynamic way with a range of cytoskeletal proteins. As cells rounded up after β-catenin knockdown, a functional relationship between β-catenin and cell structure is evident. As shown in Figure 2A, a marked reduction of cell area is observed after β-catenin knockdown. Data showed that cellular area was significantly reduced in the cytosolic but not in nuclear fraction (Figure 2B). Main molecular actors associated with cell area perturbations in the cytosol may include cytoskeletal proteins, and actin regulatory proteins. Given the observed alterations in cell morphology, to characterize the response of different cytoskeletal components on β-catenin induced cell modifications, we performed TRITC-conjugated phalloidin staining to visualize the actin cytoskeleton and Alexa Fluor 488 α-Tubulin staining to visualize α-Tubulin organization. Analysis of microtubules and F-actin distribution, revealed a different organization of actin stress fibers in shβcat cells (Figures 2, 3). In shCTR cells, F-actin filaments are predominantly organized in peripheral bundles. By contrast, in shβcat cells F-actin filaments were assembled into thick actin stress fibers organized as transverse arcs crossing the cell surface (Figures 3A,B and zoom area). We presume that this phenotype is a functional consequence of actin filaments severing mediated by actin binding proteins. To test whether this actin organization was correlated with the activation status of the actin regulator Cofilin (Cof), we determined the expression status of Cof by western blotting analysis. After β-catenin knockdown, we observed a significant lower phosphorylation status of Cof (Figure 2C), suggesting protein activation and enhanced cytoskeletal dynamics in response to β-catenin-associated cellular modifications. On the other hand, the organization of α-Tubulin filaments network did not appear significantly modified after β-catenin knockdown. As shown in Figure 2A, microtubules form meshwork-like structures with no striking difference observed between the two cell lines. This means that changes in cell shape after β-catenin knockdown corresponded predominantly with reorganization of F-actin and altered expression of actin binding proteins, and not directly related to changes in the organization of α-Tubulin filaments.

### Label free LC-MS/MS analysis of shβcat and shCTR cells

To investigate the proteomic differences associated with β-catenin knockdown, whole protein extracts of three independent samples were trypsin digested by FASP and analyzed by high-resolution mass spectrometer (Q-Exactive), followed by label-free quantification. In detail, MaxQuant and Perseus analysis identified overall 2,358 common proteins between shCTR and shβcat sample groups. GO analysis by PANTHER classification system (http://pantherdb.org) was used to perform a broad functional classification of these proteins. Two major classes of biological functions were identified, represented for the 48.5% by metabolic processes (GO:0008152) and for the 46.2% by cellular process (GO:0009787). This latter group encompassed a larger subgroup of distinct biological functions including cell communication (GO:0007154), cell cycle (GO:0007049), cell proliferation (GO:0008283), cell recognition (GO:0008037), cellular component movement (GO:0006928), chromosome segregation (GO:0007059), and cytokinesis (GO:0000910) (Table 2). Overall, this confirms the biological validity of our dataset thus providing a global view of multiple processes that may be modulated after β-catenin knockdown.

**Table 2:** Panther classification system of MCF-7 identified proteins.

**GO Biological process**	**GO Cellular processes**
Biological adhesion (GO:022610) (1.1%) Biological regulation (GO:0065007) (6.2%) Cellular component organization or biogenesis (GO:0071840) (16.3%) Cellular process (GO:0009987) (46.2%) Developmental process (GO:0032502) (5.1%) Growth (GO:0040007) (0.0%) Immune system process (GO:0002376) (2.7%) Localization (GO:0051179) (14%) Locomotion (GO:0040011) (0.3%) Metabolic process (GO:0008152) (48.5%) Multicellular organismal process (GO:0032501) (4.1%) Reproduction (GO:0000003) (0.8%) Response to stimulus (GO: 0050896) (6.7%)	Cell communication (GO:0007154) (17.6%) Cell cycle (GO:0007049) (13.6%) Cell proliferation (GO:0008283) (0.6%) Cell recognition (GO:0008037) (0.1%) Cellular component movement (GO:0006928) (4.5%) Chromosome segregation (GO:0007059) (2.1%) Cytokinesis (GO:0000910) (1.8%)

*Numbers in brackets are read as: category names (accession) percentage of gene hit against total genes*.

PANTHER's over-representation statistic test was employed to calculate over- or under-represented protein classes of our MS/MS data compared to a reference list of all human genes (Human Genome). These results are shown in Table 3. As a functional consequence of the experimental approach that we used to prepare our cellular sample for LC-MS/MS analysis, PANTHER statistically calculated protein classes that are reduced or enriched in our input list; this means classes with a lower or higher number of members compared to the genes present in our reference list. More in detail, we obtained a down-regulation of specific protein classes including transcription factor, receptor, signaling molecule, and cell adhesion molecule. On the other hand, we obtained an over-representation of aminoacyl-tRNA synthetase, translation initiation factor, ribosomal protein, actin and microtubule family cytoskeletal protein classes. We believe that the FASP analysis of whole cell lysates that we performed without any step of pre-fractionation, resulted in a decreased annotation of signaling and membrane proteins, characterized by lower expression levels compared to other enriched cytosolic protein classes.

**Table 3:** PANTHER Protein class overrepresentation test (release 20170413).

**Homo sapiens (REF) PANTHER protein class**	**MCF-7 dataset fold enrichment**	**+/−**	***P*-value**
aminoacyl-tRNA synthetase	5.70	+	5.81E-07
vesicle coat protein	5.23	+	1.18E-08
chaperonin	4.69	+	3.09E-03
translation initiation factor	4.57	+	1.93E-10
translation factor	4.19	+	1.12E-13
ribonucleoprotein	4.18	+	5.22E-07
ribosomal protein	3.75	+	2.10E-24
translation elongation factor	3.52	+	1.42E-03
mRNA splicing factor	3.45	+	1.17E-10
RNA binding protein	3.20	+	1.56E-69
dehydrogenase	3.16	+	4.45E-18
mRNA processing factor	3.15	+	4.69E-12
nucleotidyltransferase	3.00	+	1.13E-03
chaperone	2.95	+	1.20E-10
reductase	2.93	+	9.51E-11
histone	2.73	+	3.17E-04
RNA helicase	2.72	+	1.58E-03
non-motor actin binding protein	2.56	+	6.68E-07
isomerase	2.45	+	3.70E-05
oxidoreductase	2.35	+	1.92E-19
lyase	2.26	+	1.09E-03
small GTPase	2.15	+	1.50E-02
membrane traffic protein	2.13	+	2.43E-08
actin family cytoskeletal protein	2.08	+	4.45E-08
ligase	2.08	+	1.16E-07
G-protein	2.06	+	4.60E-04
cytoskeletal protein	1.85	+	5.99E-11
microtubule family cytoskeletal protein	1.80	+	4.35E-02
nucleic acid binding	1.80	+	6.09E-34
transferase	1.59	+	1.74E-08
hydrolase	1.33	+	2.35E-03
transcription factor	0.47	–	1.39E-12
receptor	0.38	–	3.62E-13
signaling molecule	0.37	–	5.87E-14
serine protease	0.34	–	1.83E-02
cell adhesion molecule	0.31	–	3.69E-07
extracellular matrix protein	0.30	–	2.55E-05
defense/immunity protein	<0.2	–	8.51E-13
zinc finger transcription factor	<0.2	–	1.08E-10
G-protein coupled receptor	<0.2	–	2.99E-11
immunoglobulin superfamily cell adhesion molecule	<0.2	–	2.12E-02
membrane-bound signaling molecule	<0.2	–	5.52E-07
immunoglobulin receptor superfamily	<0.2	–	1.95E-07
cytokine	<0.2	–	3.57E-07
KRAB box transcription factor	<0.2	–	3.41E-09

*MCF-7 MS/MS data list is compared to the Homo sapiens reference list using the binomial test. The first column contains the name of the protein class category, the second column shows the Fold Enrichment of the genes observed in the MCF-7 MS/MS data list over the expected, the third column has either + or – (under – or over-representation of this category MCF-7 MS/MS data list), the fourth column is the p–values as determined by the binomial statistic. A cutoff of 0.05 was used as a starting point*.

**Figure 4:**
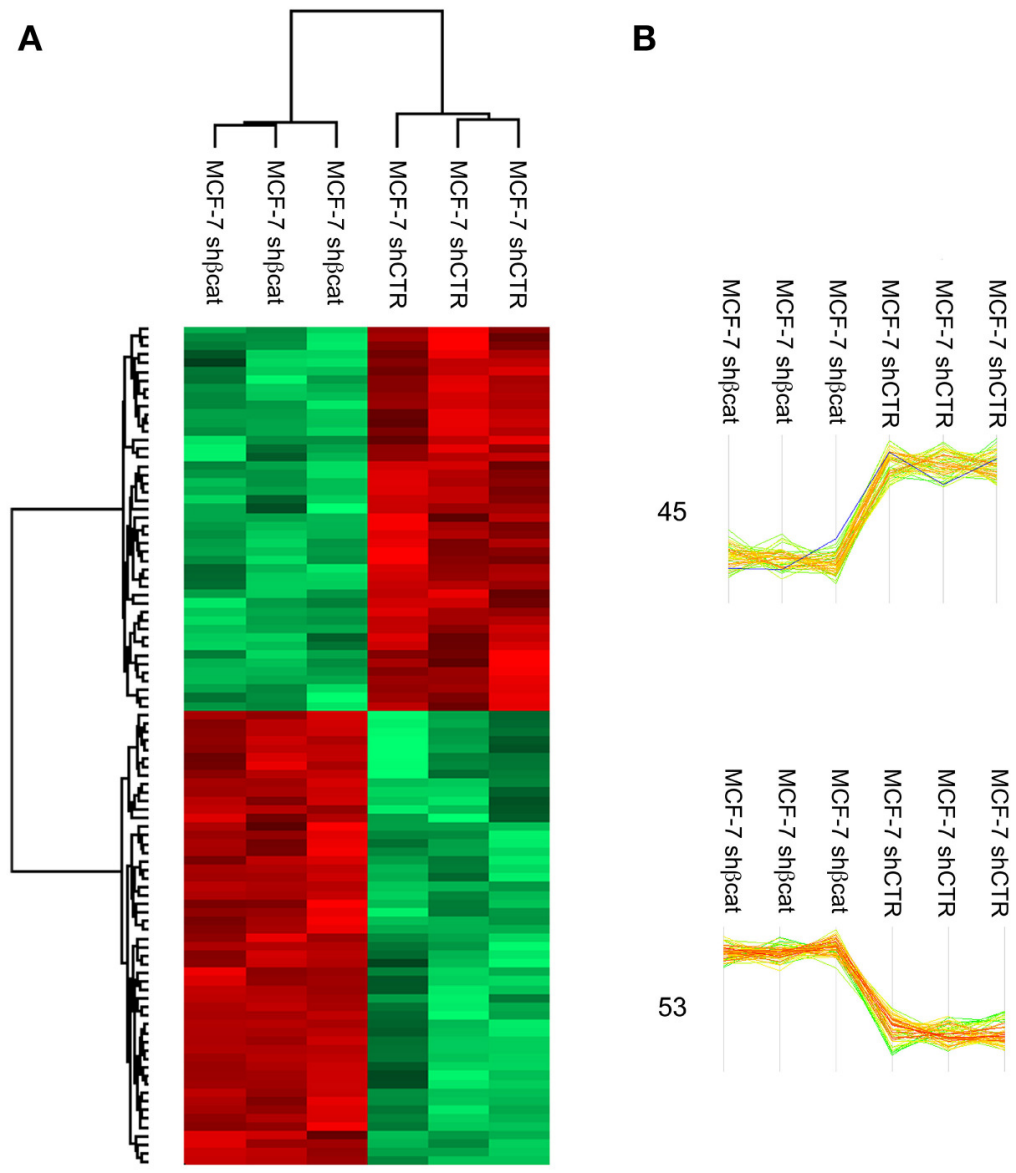
LC-MS/MS analysis of β-catenin knockdown in MCF-7 cells. **(A)** Hierarchical clustering of genes among MCF-7 shCTR and MCF-7 shβcat cells. Heat map illustrating differential expression of 98 proteins in MCF-7 shCTR cell respect to MCF-7 shβcat for biological triplicate samples. Color scale ranges from red to green (highest to lowest relative expression). **(B)** Two main clusters extracted from **(A)**. The windows contain the expression profiles of the proteins within clusters. β-catenin expression levels are highlighted in blue. The number of differentially expressed proteins in each cluster is also depicted.

MS data from shCTR and shβcat sample groups were then subjected to statistical analysis using Persues program. The heat-map generated by Perseus, segregated samples into two separated branches characterized by two clusters of up- and down-regulated proteins (Figures 4A,B). In detail, we identified a total of 98 proteins differentially expressed between shβcat and shCTR (Figures 4A,B), including 53 up-regulated and 45 down-regulated, that were quantified in at least two of three samples and that passed an multiple-sample based test for statistically significant up- or down-regulation, with a false discovery rate (FDR) of 1%. Cluster 1 and 2 of differentially expressed proteins are listed in Table 4. Cluster 1 contained proteins that are decreased after β-catenin knockdown. Importantly, β-catenin was identified among this cluster of proteins, thus providing an internal validation of our MS dataset. Cluster 2 contained proteins that are increased relative to shCTR (Figure 4B).

**Table 4:** List of differentially expressed proteins in MCF-7 shβcat cells respect to MCF-7 shCTR.

**Gene names**	**Protein names**	**Uniprot protein IDs**	**Peptides**	**Sequence coverage**
**CLUSTER 1 DOWN-REGULATED PROTEINS**				
PDHA1	Pyruvate dehydrogenase E1 component subunit alpha, somatic form, mitochondrial	P08559	12	39.7
TXNRD1	Thioredoxin reductase 1, cytoplasmic	Q16881	22	60.6
FLNB	Filamin-B	B2ZZ83	146	74.6
PDIA6	Protein disulfide-isomerase A6	Q15084	15	42.6
PITRM1	Presequence protease, mitochondrial	Q5JRX3	29	39.4
G6PD	Glucose-6-phosphate 1-dehydrogenase	E7EUI8	23	79.4
SLC3A2	4F2 cell-surface antigen heavy chain	P08195	23	45.9
PYGL	Glycogen phosphorylase, liver form	P06737	28	45.3
DDX39A	ATP-dependent RNA helicase	O00148	18	53.9
HAX1	HCLS1-associated protein X-1	O00165	2	16.9
BCAT2	Branched-chain-amino-acid aminotransferase, mitochondrial	O15382	15	47.4
IPO8	Importin-8	O15397	4	4.4
ACTN4	Alpha-actinin-4	O43707	64	76
SF3B1	Splicing factor 3B subunit 1	O75533	37	42.6
CLPX	ATP-dependent Clp protease ATP-binding subunit clpX-like, mitochondrial	O76031	14	27
DLAT	Dihydrolipoyllysine-residue acetyltransferase component of pyruvate dehydrogenase complex, mitochondrial	P10515	8	15.5
HSPD1	60 kDa heat shock protein, mitochondrial	P10809	41	75
PDIA4	Protein disulfide-isomerase A4	P13667	28	51
PLS3	Plastin-3	P13797	35	76.8
NDUFS1	NADH-ubiquinone oxidoreductase 75 kDa subunit, mitochondrial	P28331	19	37.8
CTNNB1	Catenin beta-1	P35222	17	29.1
PPP1CA	Serine/threonine-protein phosphatase PP1-alpha catalytic subunit	P62136	24	67.3
MRPS35	28S ribosomal protein S35, mitochondrial	P82673	5	21.1
LACTB	Serine beta-lactamase-like protein LACTB, mitochondrial	P83111	10	23.8
OGDH	2-oxoglutarate dehydrogenase, mitochondrial	Q02218	32	45.8
SMARCB1	SWI/SNF-related matrix-associated actin-dependent regulator of chromatin subfamily B member 1	Q12824	3	16.8
LMAN2	Vesicular integral-membrane protein VIP36	Q12907	14	51.1
TRIM28	Transcription intermediary factor 1-beta	Q13263	33	62.5
GANAB	Neutral alpha-glucosidase AB	Q14697	41	55.6
ATP6AP1	V-type proton ATPase subunit S1	Q15904	6	16
SLC25A24	Calcium-binding mitochondrial carrier protein SCaMC-1	Q6NUK1	21	59.7
CDC73	Parafibromin	Q6P1J9	7	14.3
DHX30	Putative ATP-dependent RNA helicase	Q7L2E3	21	22.8
RBM45	RNA-binding protein 45	Q8IUH3	3	8
LPCAT1	Lysophosphatidylcholine acyltransferase 1	Q8NF37	15	31.5
BRIX1	Ribosome biogenesis protein BRX1 homolog	Q8TDN6	13	48.7
DDX17	Probable ATP-dependent RNA helicase	Q92841	30	49.1
ERO1L	ERO1-like protein alpha	Q96HE7	18	52.6
POP1	Ribonucleases P/MRP protein subunit POP1	Q99575	14	18
DDX47	Probable ATP-dependent RNA helicase DDX47	Q9H0S4	9	26.4
NANS	Sialic acid synthase	Q9NR45	12	49.3
MYOF	Myoferlin	Q9NZM1	88	52.6
UBQLN2	Ubiquilin-2	Q9UHD9	8	17.5
DRG1	Developmentally-regulated GTP-binding protein 1	Q9Y295	11	40.9
RCL1	RNA 3′-terminal phosphate cyclase-like protein	Q9Y2P8	2	4.3
**CLUSTER 2 UP-REGULATED PROTEINS**				
KIAA1598	Shootin-Shootin-1	A0MZ66	7	17
RBM39	RNA-binding protein 39	Q14498	12	33.5
NUDT14	Uridine diphosphate glucose pyrophosphatase	O95848	2	17.4
DHRS2	Dehydrogenase/reductase SDR family member 2, mitochondrial	Q13268	4	24.2
SRP19	Signal recognition particle 19 kDa protein	P09132	2	25.7
LRBA	Lipopolysaccharide-responsive and beige-like anchor protein	P50851	11	5.4
NUCB2	Nucleobindin-2	P80303	6	24
NPTN	Neuroplastin	Q9Y639	4	12.4
ARPC4	Actin-related protein 2/3 complex subunit 4	P59998	8	51.4
SCAMP1	Secretory carrier-associated membrane protein 1	O15126	4	21.6
ATP5H	ATP synthase subunit d, mitochondrial	O75947	6	44.1
TRIM16	Tripartite motif-containing protein 16	O95361	7	17.2
FTH1	Ferritin heavy chain	P02794	4	24.6
TPM3	Tropomyosin alpha-3 chain	P06753	10	35.8
PFKM	ATP-dependent 6-phosphofructokinase, muscle type	P08237	12	20.4
ADH5	Alcohol dehydrogenase class-3	P11766	10	33.4
CAPN2	Calpain-2 catalytic subunit	P17655	28	52
CRABP2	Cellular retinoic acid-binding protein 2	P29373	6	41.3
BLVRA	Biliverdin reductase A	P53004	11	46.3
NAPA	Alpha-soluble NSF attachment protein	P54920	14	58.3
MYL6	Myosin light polypeptide 6	P60660	9	73.5
S100A10	Protein S100-A10	P60903	3	35.1
RPS6	40S ribosomal protein S6	P62753	4	18.9
TPM4	Tropomyosin alpha-4 chain	P67936	7	32.7
SET	Protein SET	Q01105	8	50.7
NUCB1	Nucleobindin-1	Q02818	12	27.5
SMC1A	Structural maintenance of chromosomes protein 1A	Q14683	20	18.1
NUMA1	Nuclear mitotic apparatus protein 1	Q14980	54	35.6
PEA15	Astrocytic phosphoprotein PEA-15	Q15121	7	70.8
SAFB	Scaffold attachment factor B1	Q15424	9	16.6
CCDC6	Coiled-coil domain-containing protein 6	Q16204	7	17.7
FSCN1	Fascin	Q16658	16	41.2
LRRFIP1	Leucine-rich repeat flightless-interacting protein 1	Q32MZ4	10	17
MYH14	Myosin-14	Q7Z406	42	28
KTN1	Kinectin	Q86UP2	46	41.9
NMD3	60S ribosomal export protein NMD3	Q96D46	13	44.9
CHAMP1	Chromosome alignment-maintaining phosphoprotein 1	Q96JM3	3	4.2
PFDN5	Prefoldin subunit 5	Q99471	3	31.2
ANP32E	Acidic leucine-rich nuclear phosphoprotein 32 family member E	Q9BTT0	7	29.5
DDX23	Probable ATP-dependent RNA helicase DDX23	Q9BUQ8	8	11.8
SRRT	Serrate RNA effector molecule homolog	Q9BXP5	12	16.2
DCTPP1	dCTP pyrophosphatase 1	Q9H773	4	25.9
ARMT1	Protein-glutamate O-methyltransferase	Q9H993	13	45.8
LUC7L	Putative RNA-binding protein Luc7-like 1	Q9NQ29	8	27.7
CTPS2	CTP synthase 2	Q9NRF8	6	11.4
IMP3	U3 small nucleolar ribonucleoprotein protein IMP3	Q9NV31	3	23.4
SLTM	SAFB-like transcription modulator	Q9NWH9	4	5.3
RCC2	Protein RCC2	Q9P258	17	42.7
PI4KB	Phosphatidylinositol 4-kinase beta	Q9UBF8	4	6.5
TES	Testin	Q9UGI8	6	22.1
SMC3	Structural maintenance of chromosomes protein 3	Q9UQE7	22	25.2
NUDC	Nuclear migration protein nudC	Q9Y266	14	50.2
FIS1	Mitochondrial fission 1 protein	Q9Y3D6	4	34.2

**Figure 5:**
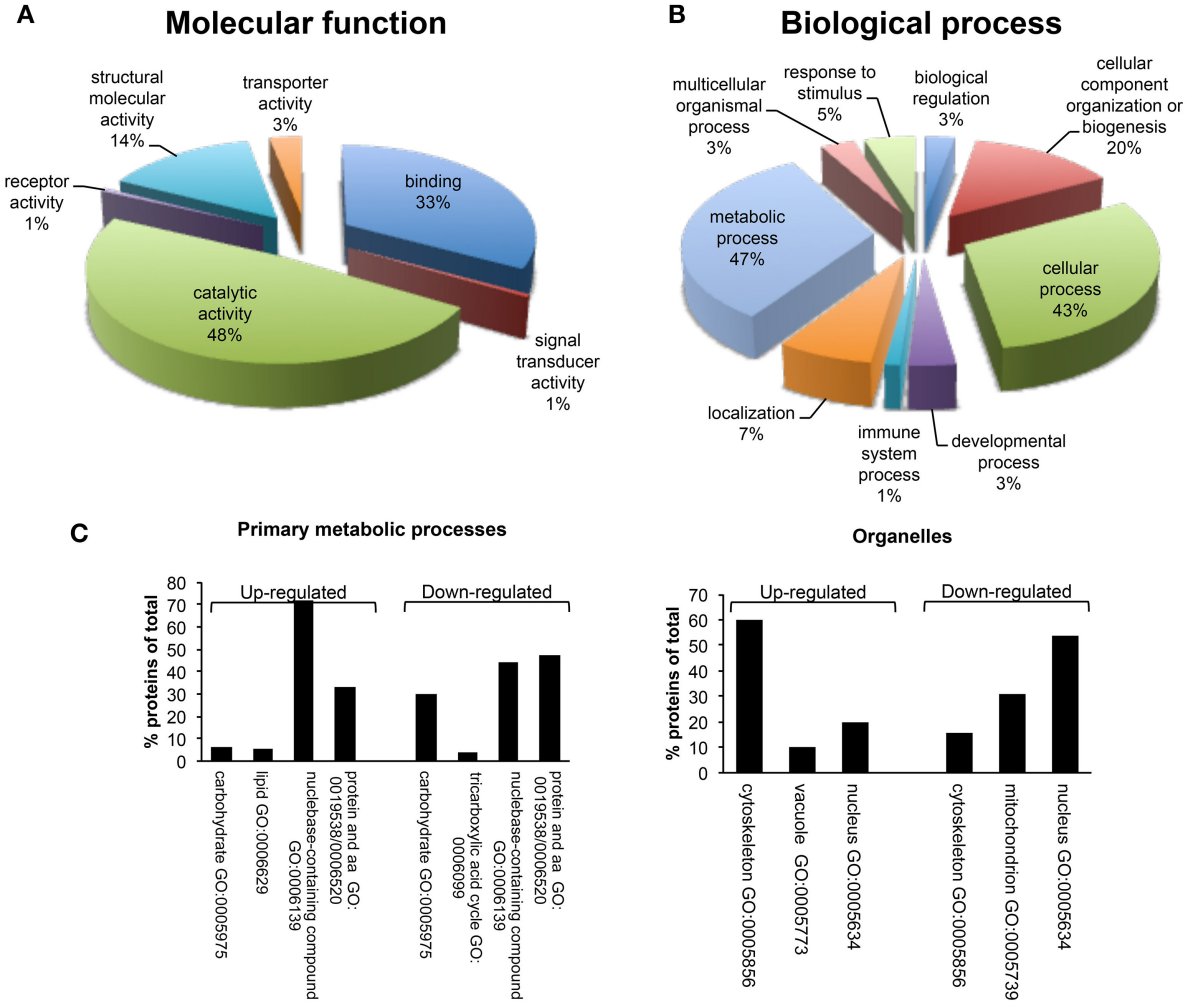
Gene ontology analysis using PANTHER. **(A)** Molecular function and **(B)** Biological process classes assigned to all differentially regulated proteins identified after proteomic analysis in MCF-7 shCTR and MCF-7 shβcat cells. It should be pointed that PANTHER may attribute multiple classes to a given protein. A complete list of the proteins can be found in Table 4. **(C)** Distribution among Primary metabolic processes and Organelle localization of down- and up-regulated proteins in MCF-7 shβcat respect to MCF-7 shCTR cells using PANTHER analysis.

A bioinformatics analysis of differentially expressed proteins was carried out using PANTHER. These proteins were classified according to their molecular functions and biological processes (Figure 5). Gene ontology categorization based on molecular functions identified four main classes of proteins involved in binding (33%), structural molecule activity (14%), transport activity (3%) and, the majority, in catalytic activity (48%) (Figure 5A). GO classification of dataset based on the biological process, led to the identification of 9 different biological processes, including metabolic process, localization, immune system process, developmental process, cellular process, cellular component organization or biogenesis, biological regulation, response to stimulus, and multicellular organismal process. The metabolic process group was identified as the most abundant GO biological process (47%) (Figure 5B). This category includes a set of differentially expressed proteins involved in the regulation of primary metabolic pathways, including protein metabolism, nucleobase-containing compound metabolism, lipid and carbohydrate metabolic processes, and tricarboxylic acid (TCA) cycle (Figure 5C). In detail, proteins with a role of regulation of carbohydrate metabolism and TCA cycle appeared as down-regulated after β-catenin knockdown; on the contrary, proteins that are associated with lipid metabolism were up-regulated compared to shCTR cells. This marks a specific metabolic modification induced in MCF-7 cells after β-catenin knockdown.

PANTHER GO analysis of subcellular localization was also performed. GO analysis indicated the common localization at nuclear and cytoskeletal level of several up- and down-regulated proteins (Figure 5C). This is in line with the morphological and functional modifications associated with β-catenin knockdown that we described above. Interestingly, down-regulated proteins showed a specific localization at mitochondria level. These results not only provide information about the spatial localization of our proteins, but also prompted us to suggest a possible modification of mitochondrial activity as reply to β-catenin induced metabolic modifications. Consistent with these observations, we decide to better characterize cellular metabolism of shβcat cells in terms of mitochondrial activity and primary metabolic processes.

### Knockdown of β-catenin reduces mitochondrial mass

To trace the mitochondrial phenotype after β-catenin knockdown, we used two different fluorescent probes to quantitate mitochondrial mass and mitochondrial membrane potential. We used a probe that is dependent on mitochondrial membrane potential to accumulate in mitochondria, MitoTracker Red CMXRos, and hence its staining correlates with mitochondrial function, and MitoTracker Green FM that is an optimal indicator of mitochondrial mass regardless of mitochondrial membrane potential.

**Figure 6:**
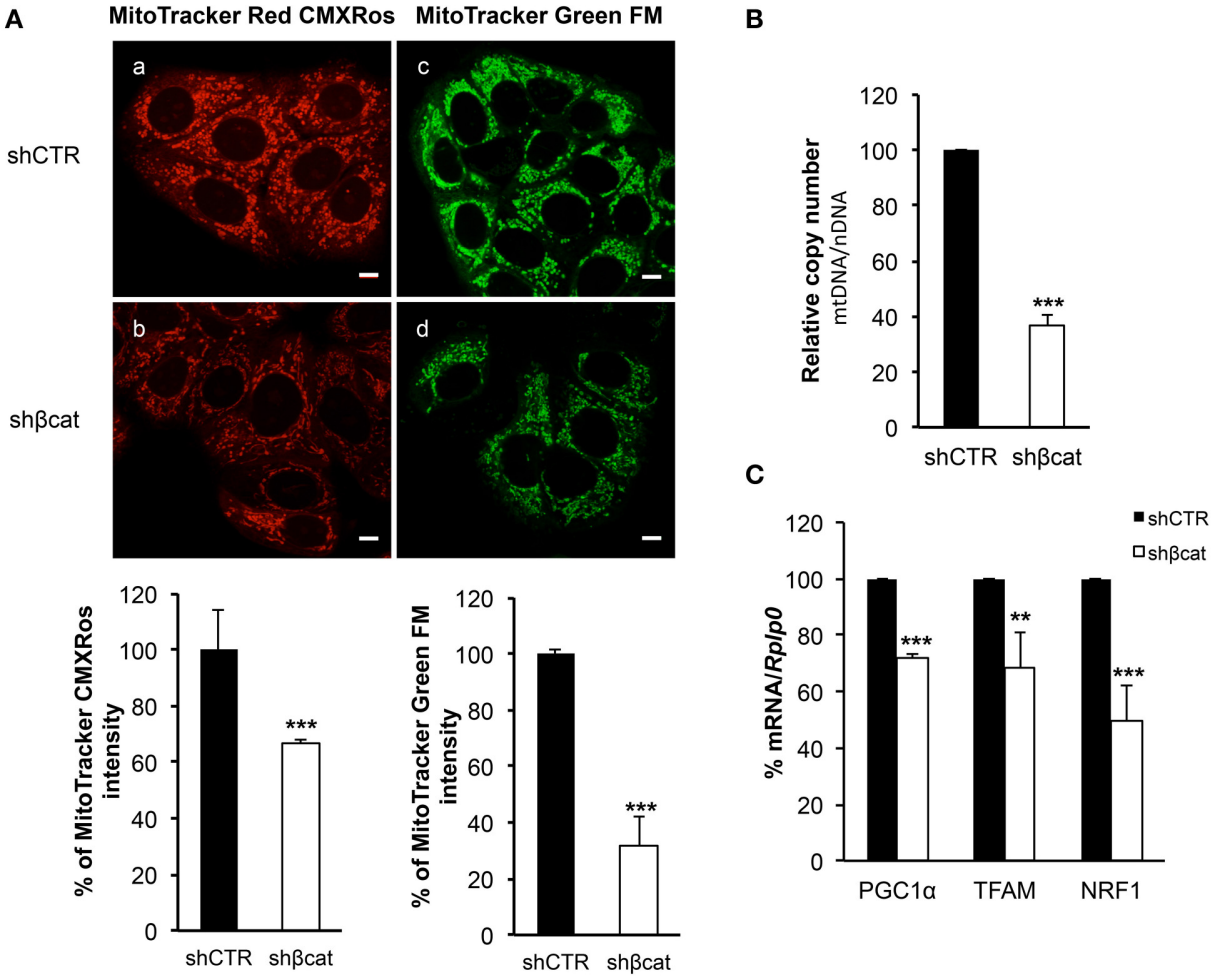
β-catenin controls mitochondrial biogenesis. **(A)** Staining in live imaging of MCF-7 shCTR and MCF-7 shβcat mitochondria. MitoTracker CMXRos (red; **a,b**) accumulation is dependent upon mitochondrial membrane potential while MitoTracker Green FM (green; **c,d**) stains mitochondria regardless membrane potential, scale bar: 10 μm. Quantification of different intensities was performed with ImageJ software and represented in histograms. Data represent the mean ± SD from 3 independent experiments, with a significant difference of **P < 0.005 and ***P < 0.001. **(B)** Quantitative Real time PCR was used to determine nuclear DNA (nDNA) and mitochondrial DNA (mtDNA) contents in MCF-7 shCTR (black columns) and MCF-7 shβcat (white columns). The mtDNA level was expressed as the ratio of mtDNA to nDNA copy number (mtDNA/nDNA). **(C)** Quantitative Real time PCR was used to determine PGC1α, TFAM and NRF1 gene expression levels in MCF-7 shCTR and MCF-7 shβcat cells. Data represent the mean ± SD from 3 independent experiments. **P < 0.005, ***P < 0.001.

As shown in Figure 6A, the distribution of green and red fluorescence was uniform in the two sections indicating that the organelles were equally dispersed in the cytoplasm. Moreover, to avoid problems caused by the decrease of fluorochromes excitation through the sample, due to increased distance of the section from the light source, we measured the signal intensity from 10 different sections per samples and integrated the values by ImageJ program. The summation of these values revealed a reduction of about 30% (*P* ≤ 0.001) in MitoTracker Red CMXRos intensity, and a reduction of about 70% (*P* ≤ 0.001) of MitoTracker Green FM staining intensity. These data allow us to speculate that the reduced MitoTracker green intensity measured in β-catenin knockout cells was correlated to the reduced mitochondrial mass.

This loss of mitochondrial mass was confirmed by analyzing the mean mtDNA/nDNA copy number by Real time PCR. Results reported in Figure 6B showed a reduction of about 60% in relative mtDNA content in knockout cells compared with controls (Figure 6B).

Having demonstrated that mitochondrial mass is reduced in shβcat cells, we next sought to determine the molecular regulation for this phenotype. To do this, we investigated the expression of peroxisome proliferator-activated receptor coactivator-1α (PGC-1α), a key transcriptional regulator of cellular energy metabolism and mitochondrial biogenesis (Liang and Ward, 2006). Moreover, we also determined the expression level of the mitochondria transcription factor A (TFAM) and nuclear respiratory factor1 (NRF1). NRF1 and TFAM have been demonstrated to regulate mitochondrial function and biogenesis in various tissues (Cam et al., 2004; Guerra et al., 2011). Real time PCR revealed a significant decrease in the expression of the three transcription factors in shβcat cells relative to shCTR (Figure 6C). Thus, shβcat cells showed a loss of mitochondrial mass that is correlated with a down-regulation of mitochondrial biogenesis transcription factors.

### β-catenin knockdown modulates lipid metabolism

**Figure 7:**
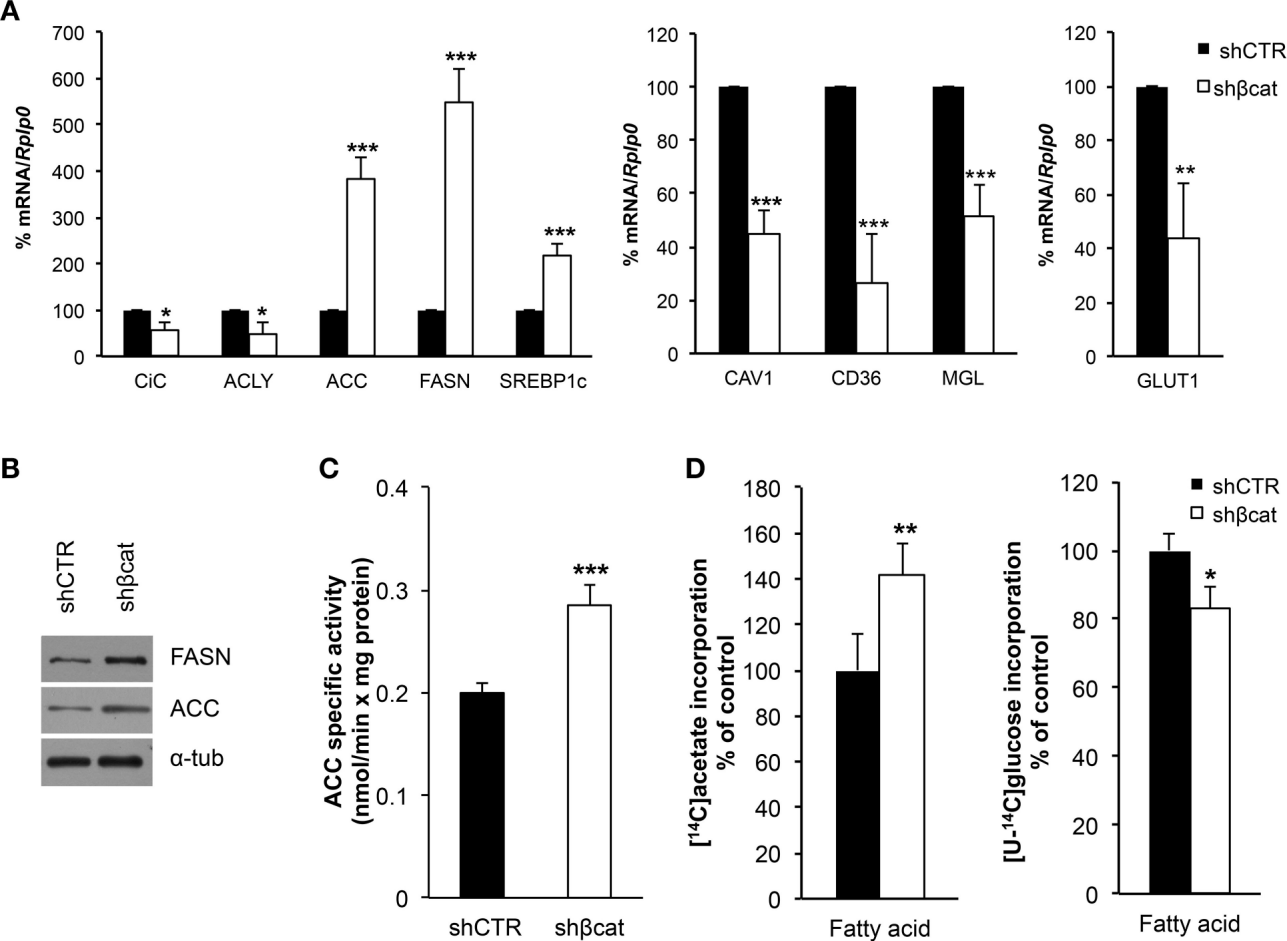
Metabolic effect of β-catenin silencing in MCF-7 cells. **(A)** Quantitative Real time PCR was used to determine mRNA expression levels of citrate carrier (CiC), ATP-citrate lyase (ACLY), acetyl-CoA carboxylase (ACC), fatty acid synthase (FASN) and Sterol Regulatory Element Binding Protein1c (SREBP1c), involved in lipogenesis; Caveolin 1 (CAV1), CD36 and monoacylglycerol lipase (MGL) involved in fatty acid transport and remodeling; and glucose transporter 1 (GLUT1) regulating glucose transport. **(B)** Western blot of FASN and Acetyl CoA Carboxylase (ACC) protein levels. α-tubulin (α-tub) was used for signal normalization. The images are representative of three independent experiments. **(C)** ACC specific activity measured in digitonin-permeabilized cells is expressed as nmoles [^14^C]Acetyl-CoA incorporated into fatty acids/min × mg protein. **(D)** [1-^14^C]acetate and [U-^14^C]glucose incorporation into fatty acids was followed by incubating cells with the labeled substrates for 1 h. After this time synthetized fatty acids were extracted and radioactivity counted. Data represent the mean ± SD from 4 independent experiments; asterisks indicate significant differences compared to shCTR. *P < 0.01, **P < 0.005, ***P < 0.001.

As discussed above, LC-MS/MS and bioinformatics analysis revealed alterations of biological processes related to primary metabolism including down-regulation of proteins belonging to carbohydrate metabolism and TCA cycle, and up-regulation of proteins regulating lipid metabolism. To gain further insight into the metabolic modifications induced after β-catenin knockdown, MS data were complemented with the analysis of a selection of genes involved in carbohydrate and lipid metabolism (Figure 7). To do this, we analyzed by Real time PCR the expression level of citrate carrier (CiC), ATP-citrate lyase (ACLY), acetyl-CoA carboxylase (ACC) and fatty acid synthase (FASN) that are involved in the *de novo* synthesis of fatty acids, CD36, Caveolin (CAV1), and monoacyl glycerol lipase (MGL), that are involved in the uptake of lipids, lipid droplets formation, and hydrolysis of monoacylglycerols, respectively. Moreover, the expression of GLUT1 was also investigated.

In basal growth conditions, MCF-7 cells are characterized by a lipogenic phenotype with high expression level of lipogenic enzymes (manuscript in preparation) including CiC, ACLY, FASN and ACC. After β-catenin knockdown, we observed a modestly reduced expression (less than 1.5 fold change) of CiC and ACLY, but a significant increase of ACC and FASN together with a parallel increased expression of Sterol Regulatory Element Binding Protein-1c (SREBP1-c), a master transcription factor regulating ACC and FASN expression (Figure 7A). We further confirm these data by performing a Western blot analysis of FASN and ACC. As shown in Figure 7B, we observed an up-regulation of both proteins in shβcat cells.

A different result emerged from the mRNA expression analysis of other metabolic genes that regulate fatty acids and glucose uptake. We found a reduced expression of CAV1 and CD36 genes in shβcat, this may result in a reduction of the uptake of lipids with a consequent lower synthesis of triacylglycerol and reduced mobilization of monoacylglycerols by MGL, whose expression resulted down-regulated after βcat knockdown. Additionally, we also observed that shβcat cells showed a reduced expression level of GLUT1 compared to shCTR cells (Figure 7A).

Enzymatic activity assay confirmed that higher ACC expression in shβcat cells was reflected in an increased ACC activity, and greater [1-^14^C]acetate incorporation into fatty acids (Figures 7C,D). To note that the synthesis of fatty acids from [U-^14^C]glucose was reduced in shβcat with respect to shCTR (Figure 7D) thus supporting a reduced entry of glucose in shβcat as highlighted by the lower expression of the glucose transporter GLUT1.

## Discussion

The role of aberrant β-catenin pathway activation in the various stages of tumorigenesis is well recognized. However, cellular functions and processes regulated by β-catenin appear to be closely related to a specific proteogenomics landscape. This is true for colon cancer cells where a cell-specific protein networks modulate Wnt signaling (Song et al., 2014), and for breast cancer where alterations of β-catenin levels drive the progression of the basal category of ErbB2-positive breast cancer (Tung et al., 2017).

Here, we report the morphological, molecular, and functional modifications induced after β-catenin knockdown in MCF-7 cells, an epithelial cellular model with a cytoplasmic and nuclear pool of β-catenin and that lack active Wnt/β-catenin signaling (Lamb et al., 2013). Consistent with what observed in luminal ErbB2 model (Tung et al., 2017), knockdown by shRNA in MCF-7 resulted in a reduction of β-catenin expression in both cellular pools. Functionally, results of our *in vitro* experiments reflect clearly the multifunctional role that β-catenin plays in this cellular model as structural protein, involved in the functional organization of cell-cell contacts, and as transcriptional factor and key mediator of Wnt signaling pathway with a prominent role as regulator of cellular metabolism.

Although epithelial structure of MCF-7 cells was not significantly affected, as cells continued to growth as clusters of closely packed cells, reduced expression of β-catenin by shRNA altered cellular morphology with a significant reduction of cell area. A mechanistic insight of this result may be found if we consider that a substantial pool of β-catenin at the plasma membrane is lost in MCF-7 cells where β-catenin had been knocked down. This cellular alteration is consistent with the role of β-catenin in regulating epithelial structure through the interaction with E-cadherin, α-catenin, and actin filaments, and correlated with modifications in actin organization and expression of actin binding proteins regulators such as Cofilin that we observed in shβcat cells. Moreover, MS/MS data suggest than when β-catenin is knocked down, a set of actin remodeling proteins is modulated and probably functionally correlated with the disturbance of β-catenin interactome at cell-cell junction. In particular, Actin-related protein 2/3 complex subunit 4, Tropomyosin alpha-3 chain, Tropomyosin alpha-4 chain and Fascin are present in the cluster 2 of up-regulated proteins, on the contrary, HCLS1-associated protein X-1, and Plastin-3 are down-regulated. Moreover, we observed changes in the expression of scaffold proteins including Filamin-B and Alpha-actinin-4 that are down-regulated in shβcat cells, and Shootin-1, Testin, Myosin light polypeptide 6, and Myosin 14 that are up-regulated. Overall, this means that the actin cytoskeleton and scaffold proteins respond to changes in β-catenin expressions and this was associated with an evident modification in cell structure. Cytoskeletal remodeling has a major impact on the migratory properties of a range of cell types. Collectively migrating cells require a functional actin-myosin contractile apparatus to drive cell movement as well as the fine regulation and recycling of membrane components including integrins. In shβcat cells, mechanisms of cell migration may be impaired due to modifications of the actin cytoskeletal structure and signaling regulators such as HCLS1-associated protein X-1 (HAX1), that plays role in regulating cell migration mediated by integrins and cell membrane receptors (Ramsay et al., 2007; Mekkawy et al., 2012). Overall, these modifications explain the reduced motility of MCF-7 shβcat under conditions that stimulate wound repair.

In addition to these cytoskeletal modifications, bioinformatics analysis of our protein dataset revealed further significant alterations in the expression of proteins involved in the regulation of primary metabolic processes, namely reduced levels of carbohydrate metabolism and TCA proteins, and increased levels of lipid metabolism proteins. In detail, MS/MS data reported a down-regulation of a component of the pyruvate dehydrogenase complex, involved in the conversion of glycolytic pyruvate into acetyl-CoA, of a subunit of the respiratory chain NADH-ubiquinone oxidoreductase complex, of 2-oxoglutarate dehydrogenase participating in the tricarboxylic acid cycle and of a subunit of the V-type proton ATP-ase. These metabolic differences are also marked by the mitochondria localization of a large group of differentially expressed proteins among shβcat and controls. This may imply a reduced production of metabolic substrates with a possible functional modification of mitochondrial activity with a consequential effect on cell physiology. Recently, this has been demonstrated for LACTB, a phosphatidylserine decarboxylase that was down-regulated in shβcat cells. LACTB, by acting on mitochondrial lipid metabolism leads to increased differentiation and reduced proliferation of breast cancer cells (Keckesova et al., 2017).

As described, we observed in shβcat cells a significant reduction of mitochondrial number, as also confirmed by the decreased relative amount of mtDNA, with a concomitant reduction of mitochondrial potential. This means that reduced mitochondrial potential is dependent on changes in mitochondrial number, and that mitochondria retain their activity in shβcat cells. Loss of mitochondrial mass is consistent with repressed expression of mitochondrial biogenesis transcriptional factors PGC1α, TFAM, and NRF1 observed in shβcat cells by Real time PCR. Our data points to β-catenin as a regulator of mitochondrial biogenesis, in agreement with a previous work that described a role of Wnt signaling in the regulation of mitochondrial biogenesis and oxidative phosphorylation gene expression (Yoon et al., 2010). However, another study revealed that this is not the case in melanoma cells, further highlighting the cellular-dependent regulation of β-catenin on mitochondria physiology (Brown et al., 2017).

In addition to the transcriptional control on mitochondrial biogenesis factors, some significant differences concerning other mitochondrial proteins were observed between shCTR and shβcat cells, including the ATP-dependent Clp protease ATP-binding subunit clpX-like, mitochondrial protein (CLPX), the 28S ribosomal protein S35, mitochondrial (MRPS35), and the Presequence protease, mitochondrial (PITRM1). CLPX and PITRM1 have a role on mitochondrial function by proteolytic degradation of proteins in different mitochondrial compartments (Quirós et al., 2015). MRPS35, together with other members of MRP family, is functionally associated with mitochondrial translation of OXPHOS protein complexes (Sotgia et al., 2012). Notably, CLPX was associated to the metabolic control of several mitochondrial metabolic pathways including TCA cycle, NDUFS1 and components of the pyruvate dehydrogenase complex proteins (Fischer et al., 2015). This is particularly intriguing as these proteins were all down-regulated after β-catenin knockdown in our cell model, proposing a not previously described mechanism of regulation of β-catenin on mitochondrial physiology through mitoproteases.

In these years, increasing evidence point to the role of Myc in the regulation of cell metabolism (Camarda et al., 2017) and mitochondrial biogenesis (Morrish and Hockenbery, 2014). Myc is a well-known β-catenin target gene and resulted down-regulated in shβcat cells. CLPX, MRP35, and PITRM1 were all previously identified as mitochondrial targets of Myc (Seitz et al., 2011). This links the down-regulation of these three proteins that we observed by MS/MS to the transcriptional control of Myc mediated by β-catenin.

Several works have demonstrated the role of Myc in the regulation of several components of the glucose metabolic pathway including GLUT1 (Osthus et al., 2000). In agreement with these data, we observed in shβcat cells a down-regulation of GLUT1 expression. As the consequence of this, the metabolic flux of glucose to promote anabolic reactions may be reduced. This is also confirmed by the lower level of radiolabeled glucose incorporation into fatty acids that we observed in shβcat cells. Moreover, this may be functionally associated with the GO analysis performed by PANTHER, that classified carbohydrate and TCA among the down-regulated processes identified in shβcat cells. If a reduced flux of glucose can impact on these primary metabolic processes, shβcat cells may reprogram other pathways to balance this metabolic demand. The carbon source for fatty acid synthesis in mammalian cells is acetyl-CoA that can be supplied by the cleavage of citrate into oxaloacetate and acetyl-CoA by ACLY. When ACLY expression is reduced, mammalian cells possess an intrinsic flexibility in their ability to acquire acetyl-CoA from different sources including acetate (Zhao et al., 2016). GLUT1, CiC, and ACLY reduced expression may result in a glucose-to-acetate metabolic switch to provide acetyl-CoA for *de novo* lipogenesis. This hypothesis is in agreement with the observation that when radiolabeled acetate is added to cell in culture, shβcat cells respond to this deficiency by increasing incorporation of this substrate into fatty acids, and up-regulating the expression of transcripts associated with fatty acids synthesis including FASN and ACC by SBREP1, which acts to drive fatty acid gene synthesis (Röhrig and Schulze, 2016). These data were corroborated by an increased ACC activity measured *in situ* by permeabilizing cell membrane with digitonin. In this picture, it remains less clear the role of CAV1, CD36, and MAGL. If MCF-7 β-catenin knockdown experienced a reduced flux of glucose and exogenous fatty acids into lipids, lipid droplets may undergo lipolysis to release fatty acids to sustain phospholipid synthesis. Surprisingly, our results showed a reduced gene expression of CAV1, CD36 and MAGL, this means a possible reduction of exogenous fatty acid intake and mobilization from triglycerides. To explain this result we propose that shβcat cells could adopt a specific metabolic profile that support *de novo* fatty acid synthesis better than lipolysis in keeping with their preferential utilization of acetate, thus minimizing the need of external fatty acids to serve as sources of cell lipids. Moreover, we think that β-catenin and Myc may have a role in the regulation of CAV1, CD36 and MAGL genes. Consistent with this was the observation that Wnt1 plays a role in the regulation of CD36 via activation of the canonical Wnt pathway (Wang et al., 2015), and that CAV expression is closely associated with Myc in prostate cancer (Yang et al., 2012). These results are in line with the ones obtained from pan-cancer datasets issued from multi-cancer Translation of the Cancer Genome Atlas (TCGA). Genetic alterations in metabolic genes associated with metastatic progression analyzed, revealed that genes involved in cellular fatty acid uptake (CAV1, CD36) and *de novo* lipogenesis (PPARA, PPARD, MLXIPL) were specifically amplified at higher frequencies in metastatic tumors (Nath and Chan, 2016). Moreover, a gene-signature (CAV1, CD36, MLXIPL, CPT1C, CYP2E1) is strongly associated with EMT program across multiple cancers (Nath and Chan, 2016). These data indicate that deranged lipid metabolism may confer pro-metastatic traits and accelerate the metastatic dissemination process of cancer.

In conclusion, we present a label free proteomic analysis of breast cancer cells knocked down for β-catenin expression. These data provide molecular insights about the network of regulation of this protein, and reinforce the role of β-catenin as regulator of cell metabolism through the transcriptional control of Myc and its target genes. To note that, the activation of FASN and ACC in a condition in which the protooncogene Myc was down-regulated, disconnects *de novo* lipogenesis from the direct control by Myc through a metabolic reprogramming that supports lipid synthesis from other energetic substrates.

## Author contributions

DV designed experiments. MM and CB obtained funding. MM supervised the entire study. ES and SD performed qPCR experiments. FG performed mitochondria analysis under the supervision of CB, MT, and PS performed the knockdown experiments. AMG performed biochemical assays. JF and DV performed sample preparation and LC-MS/MS analysis under the supervision of IF. MS, PP, and AG performed confocal microscopy analysis. DV, ES, and AMG wrote the paper with input from the other authors. All authors read and approved the final manuscript.

### Conflict of interest statement

The authors declare that the research was conducted in the absence of any commercial or financial relationships that could be construed as a potential conflict of interest.
